# Hsa_circ_0079480 promotes tumor progression in acute myeloid leukemia via miR-654-3p/HDGF axis

**DOI:** 10.18632/aging.202240

**Published:** 2020-12-03

**Authors:** Qingzhu Hu, Yueli Gu, Shuxia Chen, Ying Tian, Shuo Yang

**Affiliations:** 1 Department of Hematology, The First People’s Hospital of Shangqiu, Shangqiu 476100, Henan, China

**Keywords:** acute myeloid leukemia, hsa_circ_0079480, miR-654-3p, HDGF

## Abstract

Circular RNAs (circRNAs) are newly-discovered endogenous non-coding RNAs that have vital functions in regulating gene expression in tumorigenesis. Nonetheless, the function of circRNAs in acute myeloid leukemia (AML) are not yet clarified. In this analysis, hsa_circ_0079480, a novel circRNA, has been identified as being highly expressed in AML. Loss-of-function assays showed that reduction of hsa_circ_0079480 decreased the growth and stimulated apoptosis of AML cells *in vitro*. Furthermore, miR-654-3p was sponged by hsa_circ_0079480, and hepatoma-derived growth factor (HDGF) was targeted by miR-654-3p with respect to the fundamental mechanism. Moreover, the influence on growth and apoptosis of AML cells stimulated by hsa_circ_0079480 inhibition can be rescued by miR-654-3p inhibitor or HDGF overexpression. In summary, hsa_circ_0079480 is highly expressed in AML and drives by tumor progression via regulation of hsa_circ_0079480/miR-654-3p/HDGF axis, indicating that hsa_circ_0079480 may function as a new treatment target for AML therapy.

## INTRODUCTION

Acute myeloid leukemia (AML) is a malignant cancer of the hematopoietic system, and is characterized by growth of hematopoietic cells located in the bone marrow (BM). It goes into the bloodstream and infiltrates various tissues and organs, leading to a series of clinical manifestations [1, 2]. AML is still the most frequently diagnosed malignant BM disease in adults, and accounts for approximately 80% of adult leukemia cases [3]. In spite of the developments in diagnosis and therapy, the 5-year overall survival (OS) among AML patients remains stubbornly low at approximately 30% [4, 5]. Thus, exploring the potential molecular mechanism of AML is crucial to improve the therapeutic effects.

Circular RNA (circRNA), a type of newly-discovered endogenous non-coding RNA (ncRNA) molecule, forms covalently closed-loop structures without a 5′ cap and 3′ polyadenylated tail [6, 7]. Accumulating data indicates that circRNAs function in diverse biological processes, including cancer initiation, chemical resistance, and immune responses [8, 9]. In addition, circRNAs function as competing endogenous RNA (ceRNA) by competitively being bound to miRNA response elements, eliminating the repressive effects of miRNAs on target mRNAs [10]. Wang et al. discovered that circ-MYBL2 plays a role of sponge for miR-361-3p, which can promote cervical cancer cells growth and invasion [11]. Li et al. Indicated hsa_circ_0002483, through targeting miR-182-5p, can enhance sensitivity of lung cancer for the taxol and suppressed lung cancer progression [12]. Chen et al. showed that circSnx5 controlled the immunogenicity of dendritic cells via the miR-544/SOCS1 pathway [13].

With more advanced high-throughput sequencing technology and bioinformatics, multiple dysregulated circRNAs have been observed in multiple cancers, such as AML. For example, Shang et al. showed that circPAN3 facilitates drug resistance as an autophagy inducer via the AMPK/mTOR pathway in AML cells [14]. Sun et al. showed that circMYBL2 regulated FLT3 translation by enlisting PTBP1 to encourage FLT3-ITD in AML development [15]. Shang et al. found that circPAN3-mediated drug resistance in AML by regulating the miR-153-5p/miR-183-5p-XIAP pathway [16]. Although there have been many studies on circRNA in AML tumorigenesis and progression, the roles of circRNAs and the underlying mechanism are yet to be determined.

In this study, the AML circRNA expression profile (GSE102686) was analyzed using GEO2R, and hsa_circ_0079480 was found to be significantly increased. Subsequently, we showed that hsa_circ_0079480 promoted growth of AML cells and induced apoptosis through modulation of the miR-654-3p/hepatoma-derived growth factor (HDGF) axis. Thus, the current findings suggested that hsa_circ_0079480 have the potential in AML treatment.

## RESULTS

### Hsa_circ_0079480 expressions in AML

**Figure 1:**
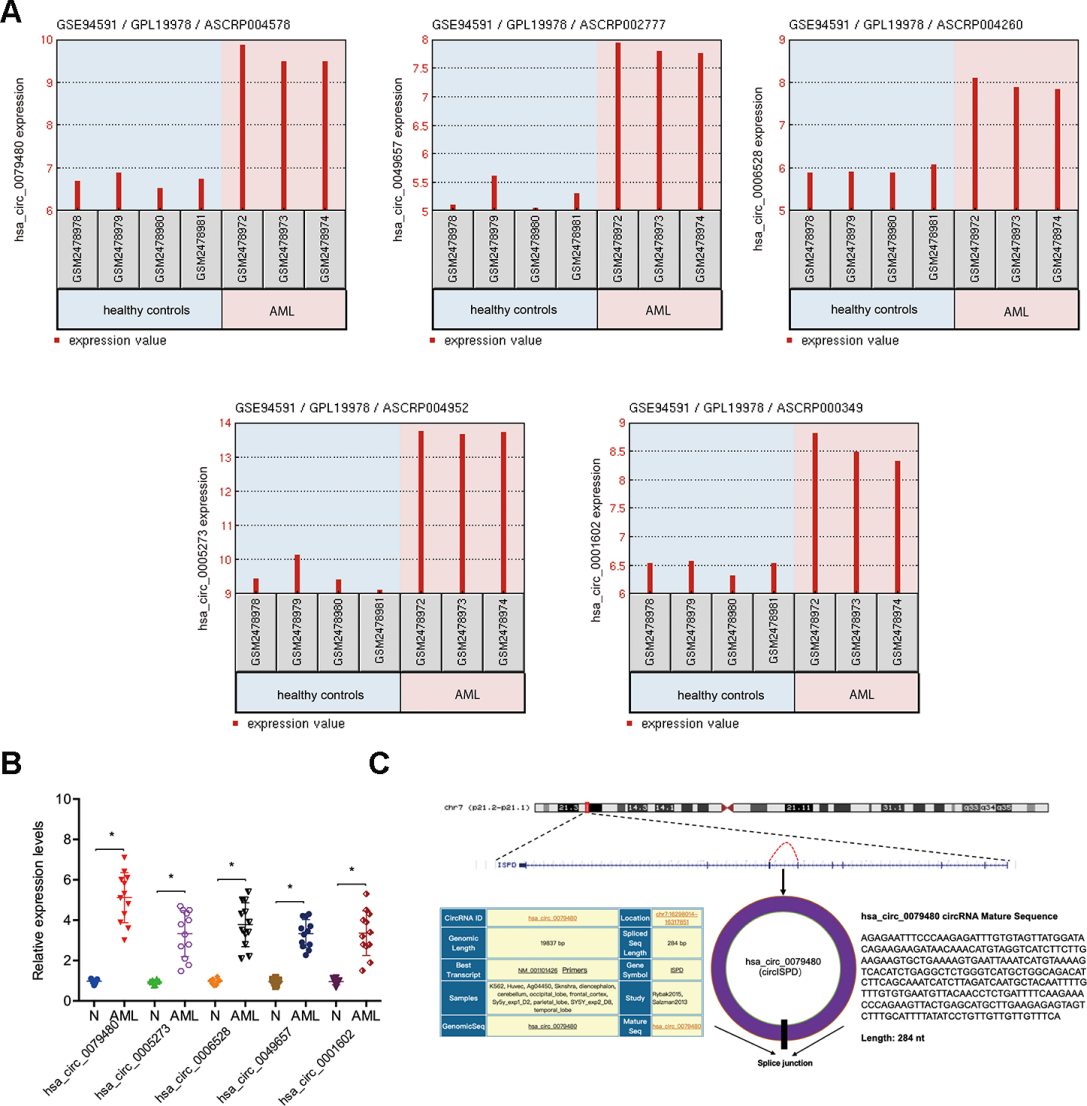
**Characteristics of hsa_circ_0079480 in AML.** (**A**) Top 5 upregulated circRNAs (hsa_circ_0079480, hsa_circ_0049657, hsa_circ_0006528, hsa_circ_0005273, and hsa_circ_0001602) in GSE94591. (**B**) The expression of 5 circRNAs expression in 12 BM samples from AML patients. (**C**) Schematic of hsa_circ_0079480. AML: acute myeloid leukemia; N: negative control. *P<0.05.

The circRNA (GSE94591) expression profile was assessed by GEO2R to find out the role of circRNAs in AML and the top 5 upregulated circRNAs (hsa_circ_0079480, hsa_circ_0049657, hsa_circ_0006528; hsa_circ_0005273, and hsa_circ_0001602) (Figure 1A) were studied. Afterwards, expressions of 5 circRNAs in 12 BM samples from AML patients were explored. Results of qRT-PCR indicated that in BM samples, expression of hsa_circ_0079480 was maximally upregulated among the 5 circRNAs (Figure 1B). Hsa_circ_0079480 is located in chr7:16298014-16317851, which is spliced from the *ISPD* gene, which the ultimate length of 284 nt (Figure 1C).

**Figure 2:**
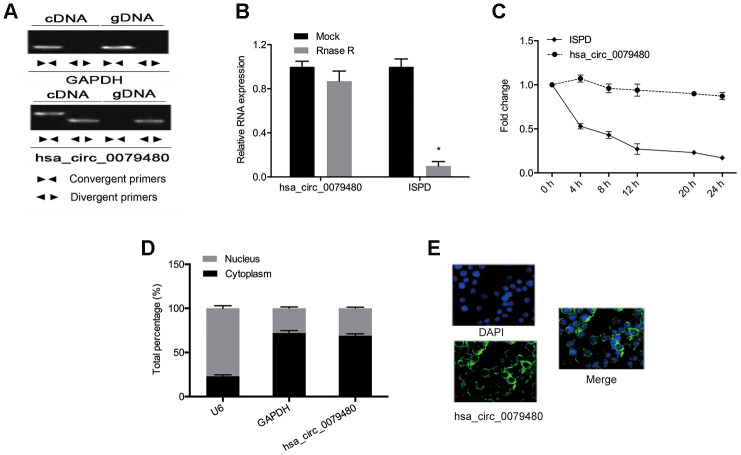
**High levels of hsa_circ_0079480 in AML.** (**A**) qRT-PCR with random hexamer primers and oligo (dT) 18 primers were utilized to identify the expression of hsa_circ_0079480 and linear *ISPD*. (**B**) The stability of hsa_circ_0079480 expression was examined by the RNase R experiment. (**C**) qRT-PCR of hsa_circ_0079480 and *ISPD* mRNA after Actinomycin D treatment. (**D**, **E**) hsa_circ_0079480 expression was measured through qRT-PCR and RNA fluorescence *in situ* hybridization (FISH) assays. *P<0.05.

Next, the circular structure of hsa_circ_0079480 was detected, and the resistance, stability, and localization of hsa_circ_0079480 were analyzed. Also, the existence of hsa_circ_0079480 in reverse transcription products was detected utilizing random primers or oligo dT primers. Figure 2A demonstrated that hsa_circ_0079480 was essentially undetectable if oligo-dT primers were utilized (Figure 2A). RNase assay indicated that circular isoform is RNase R-resistant, while the linear isoform was reduced markedly post-RNase R treatment (Figure 2B). Actinomycin D assay results showed a significantly longer circular isoform half-life compared with that of the linear isoform in ANL cells (Figure 2C). The data demonstrated that hsa_circ_0079480 is a stable and non-degradable RNA molecule. Finally, qRT-PCR and FISH experiments were used to determine subcellular localization of hsa_circ_0079480. Data shows that hsa_circ_0079480 exists in the cytoplasm of AML cells (Figure 2D, 2E).

### Hsa_circ_0079480 knockdown suppressed AML cell proliferation

**Figure 3:**
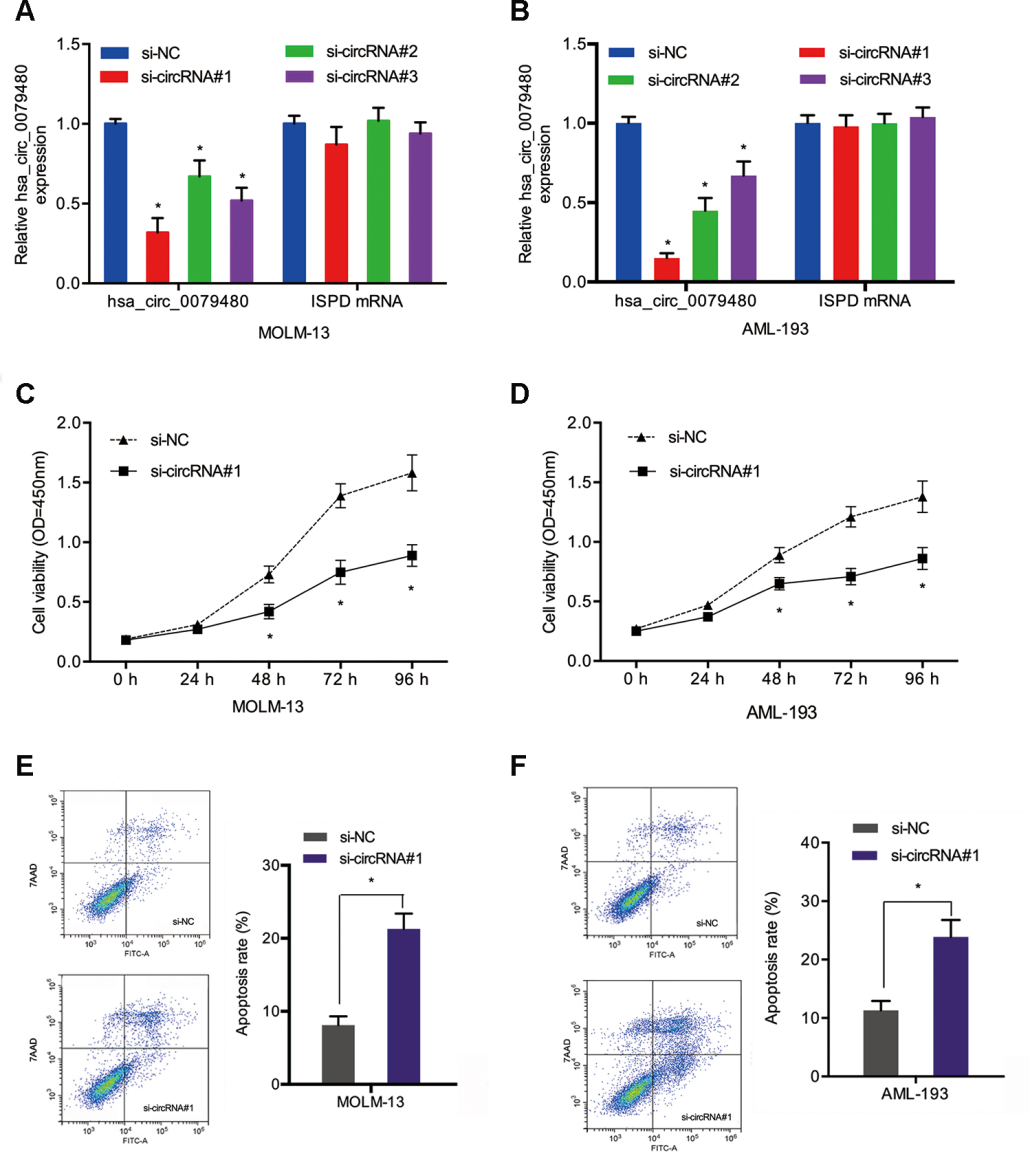
**Hsa_circ_0079480 knockdown reduced AML cell proliferation.** (**A**, **B**) The transfection efficiency of si-circ0079480 (si-circRNA) in AML cells was identified via qRT-PCR. (**C**, **D**) Cell growth was evaluated by CCK8 assays. (**E**, **F**) Cell apoptosis was determined through flow cytometry assay. *P<0.05.

Loss-of-function experiments were performed by transfection of si-circ0079480 (si-circRNA) was into AML cells to identify the oncogenic role of hsa_circ_0079480 in AML. Additionally, qRT-PCR was carried out to identify the knockdown efficiency of si-circRNA (Figure 3A, 3B). CCK-8 assay demonstrated that viability of MOLM-13 and AML-193 cells was lower in si-circRNA knockdown group in comparison to the controls (si-NC) (Figure 3C, 3D). From flow cytometry assay we can see the AML cells apoptosis rate was markedly increased by hsa_circ_0079480 knockdown (Figure 3E, 3F). The data confirmed the tumor promoter functions of hsa_circ_0079480 in AML advancement.

### Hsa_circ_0079480 played a sponge role in miR-654-3p

**Figure 4:**
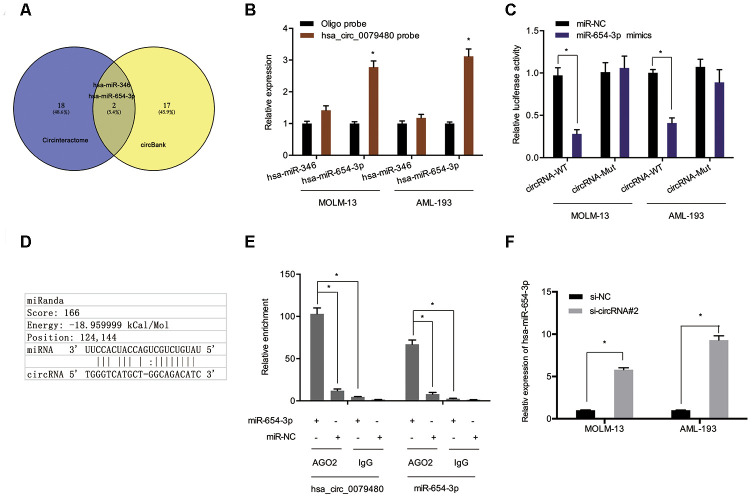
**Hsa_circ_0079480 served as sponge for miR-654-3p.** (**A**) Two potential target miRNAs (miR-346 and miR-654-3p) of hsa_circ_0079480 were predicted by circBank and CircInteractome. (**B**) Pull-down assay explored the relative expression of two miRNAs in AML cells lysates. (**D**) Schematic of the predicted binding site in hsa_circ_0079480. (**C**, **E**) The correlation among hsa_circ_0079480 and miR-654-3p was explored through luciferase reporter and RIP assays. (**F**) Hsa_circ_0079480 knockdown increased miR-654-3p levels in AML cells. *P<0.05.

To assess the molecular mechanism of hsa_circ_0079480 in AML, the miRNA targets were predicted using online tools (Circular RNA Interactome and circBank [17–20]), and two potential candidates (hsa-miR-346 and hsa-miR-654-3p) were identified (Figure 4A). Pull-down experiments validated the enrichment of hsa_circ_0079480 and miR-654-3p in both MOLM-13 and AML-193 cell lines (Figure 4B). Luciferase reporter assay indicated that upregulation of miR-654-3p reduced the luciferase activity of the hsa_circ_0079480-Wt group rather than the hsa_circ_0079480-Mut group, indicating the relationship among miR-654-3p and hsa_circ_0079480 directly (Figure 4C, 4D). RIP assay further verified the direct interaction among hsa_circ_0079480 and miR-654-3p (Figure 4E). In addition, an increased miR-654-3p expression was observed following hsa_circ_0079480 knockdown in AML cells (Figure 4F).

**Figure 5:**
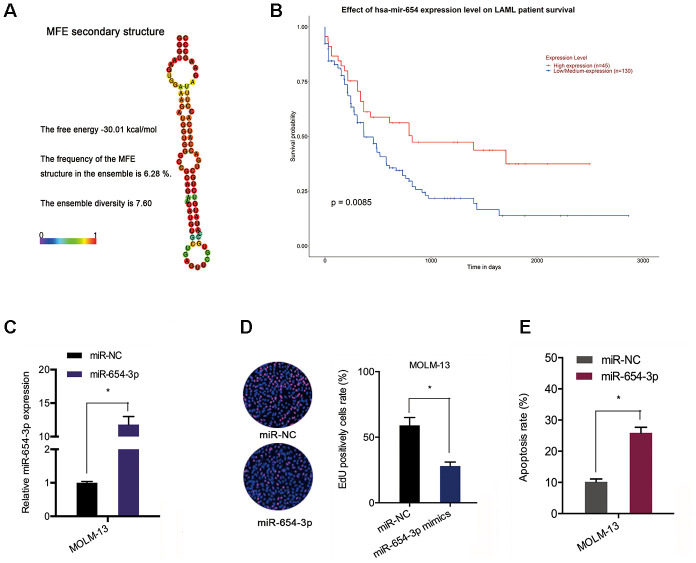
**MiR-654-3p reduced AML cell progression.** (**A**) The secondary structure of miR-654-3p. (**B**) TCGA database showed that reduced miR-654-3p levels was related to poor OS in AML patients. (**C**) The transfection efficiency of miR-654-3p mimics in AML cells. (**D**, **E**) MiR-654-3p mimics decreased AML cell growth and stimulated cell apoptosis *in vitro*. *P<0.05.

Next, the we explored the expression and potential function of miR-654-3p in AML. TCGA data indicated an association between poor OS of AML patients and reduced miR-654-3p levels (Figure 5A and 5B). Subsequently, the function of miR-654-3p in AML cells was evaluated via transfecting miR-654-3p mimics (Figure 5C). EdU assay suggested that high expression of miR-654-3p substantially hindered the proliferation of MOLM-13 cells in comparison to the negative control (Figure 5D). Apoptosis assays indicated that an increased apoptotic rate in MOLM-13 cells was associated with increased levels of miR-654-3p (Figure 5E). This data proves that hsa_circ_0079480 functions as a sponge of miR-654-3p in AML.

### MiR-654-3p directly targeted HDGF

**Figure 6:**
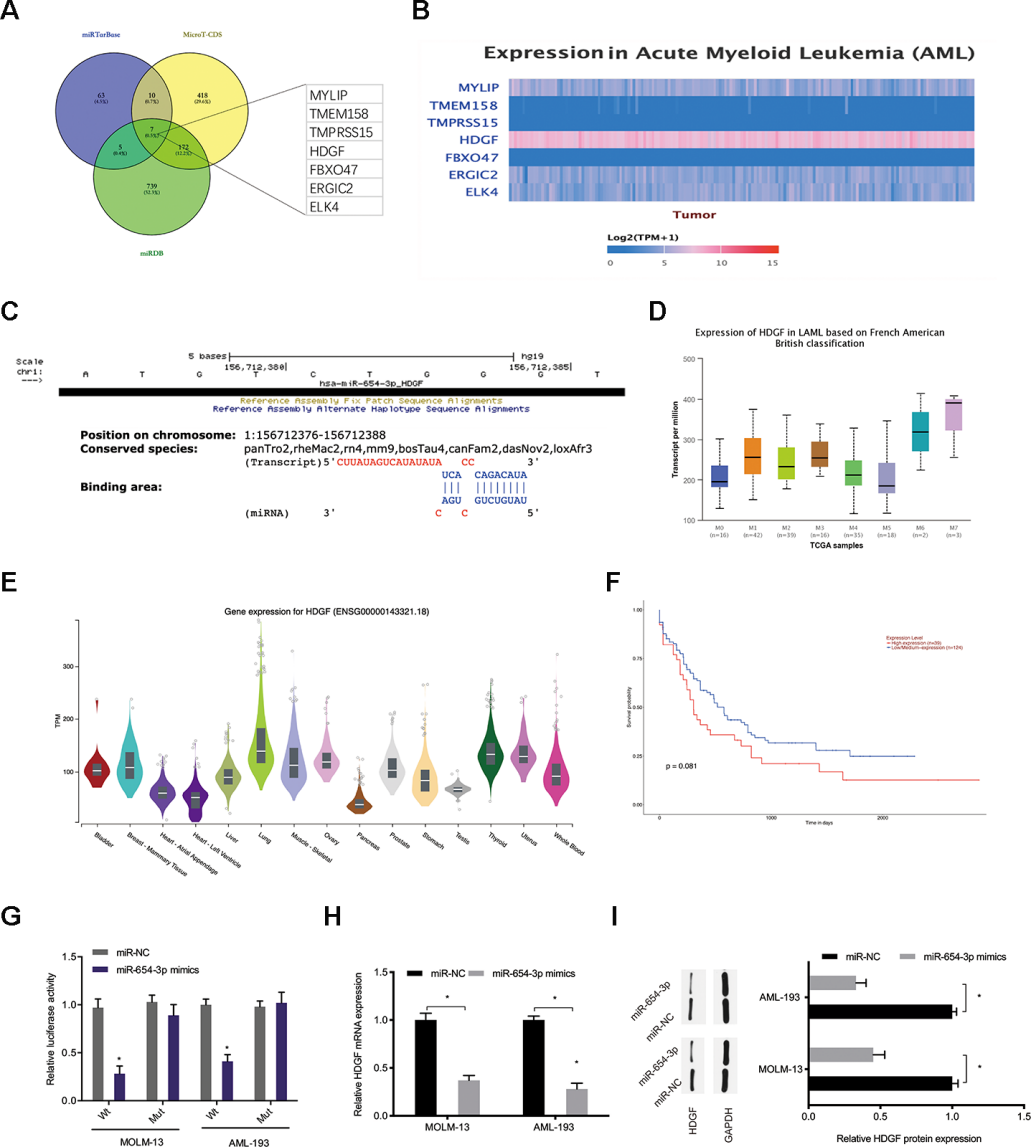
**MiR-654-3p targeted HDGF directly.** (**A**–**C**) Prediction of miR-654-3p targets by bioinformatics analysis (miRTarBase; MicroT-CDS; miRDB). (**D**) High HDGF expression in AML tissues was assessed by TCGA database. (**E**) Relative HDGF expression in normal tissues was explored by CPTAC database. (**F**) High HDGF expression was linked to poor OS in AML patients. (**G**) The correlation among miR-654-3p and HDGF was explored by luciferase reporter assays. (**H**, **I**) miR-654-3p mimics decrease the HDGF levels in AML cells at both the mRNA and protein levels. *P<0.05.

Bioinformatics analysis (miRTarBase; MicroT-CDS; miRDB [21–23]) was carried out to predict the miR-654-3p targets (Figure 6A). Among potential targets, we focused on HDGF (Figure 6B, 6C), which is known to be involved in tumorigenesis [24]. TCGA database demonstrated that HDGF levels were decreased in normal tissues and significantly increased in AML tissues (Figure 6D, 6E). High HDGF levels were related to poor OS rate in AML patients (Figure 6F). HDGF-Wt group activity was observed to be suppressed by miR-654-3p mimics through luciferase reporter assay (Figure 6G). What’s more, HDGF expression was also observed to be significantly inhibited by miR-654-3p mimics in AML cells (Figure 6H, 6I). Together, these data illustrated that miR-654-3p inversely modulated HDGF levels in AML.

### MiR-654-3p silencing (HDGF knockdown) reversed the effects of hsa_circ_0079480 suppression on AML development

**Figure 7:**
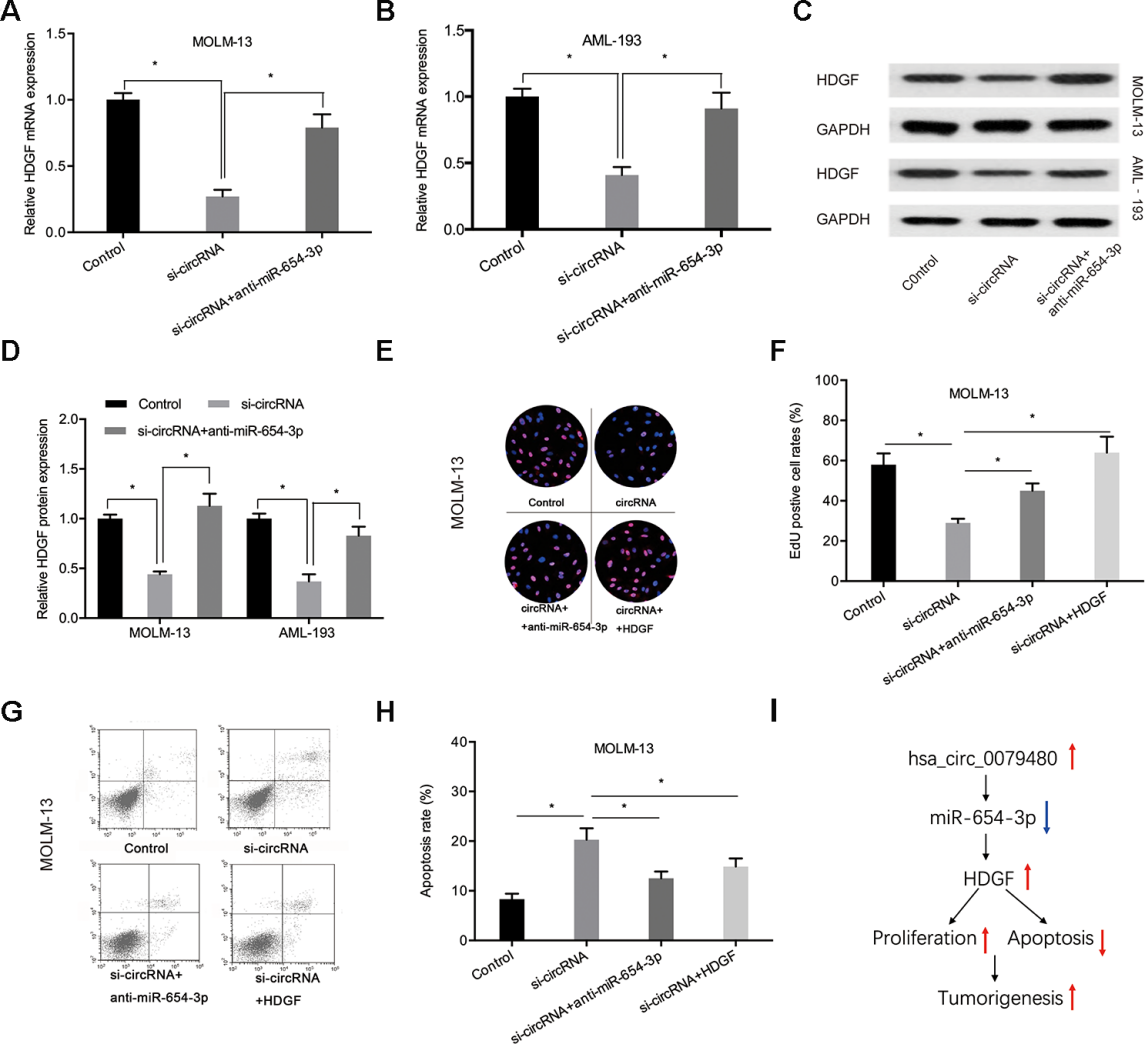
**MiR-654-3p inhibitor or HDGF overexpression rescued si-circ0079480-stimulated phenotypes in AML cells.** (**A**–**D**) MiR-654-3p inhibitor abolished the effects of si-circ0079480 on HDGF expression in AML cells. (**E**–**H**) EdU and cell apoptosis assays indicated that miR-654-3p inhibitor (or HDGF overexpression) reversed the effects of si-circ0079480 on AML progression. (**I**) Schematic of hsa_circ_0079480/miR-654-3p/HDGF in AML. *P<0.05.

In order to verify that hsa_circ_0079480 exhibits an oncogenic influence on AML progression by modulating miR-654-3p/HDGF pathway, miR-654-3p inhibitor or HDGF overexpression plasmid (HDGF) was co-transfected using si-circ0079480 (si-circRNA). qRT-PCR and Western blot assays indicated that hsa_circ_0079480 knockdown suppressed HDGF levels and miR-654-3p inhibitors reversed its role in ALM cells (Figure 7A–7D). Next, rescue assays were utilized to confirm the hsa_circ_0079480/miR-654-3p/HDGF axis in AML. EdU assay indicated that hsa_circ_0079480 suppression hindered the viability of MOLM-13 cells *in vitro*, while miR-654-3p inhibitors (or HDGF overexpression) reversed these effects (Figure 7E, 7F). Moreover, si-circ0079480-induced apoptosis of MOLM-13 cells was also rescued by a miR-654-3p inhibitor (or HDGF overexpression) (Figure 7G, 7H). Thus, these data suggested that hsa_circ_0079480 encourages AML advancement via the miR-654-3p/HDGF axis (Figure 7I).

## DISCUSSION

circRNA is an endogenous ncRNA that has attracted widespread attention owing to its unique loop structure, high stability, and conservation across evolution [25]. Accumulating data suggests that the dysregulated levels of circRNAs seen in AML exerted a crucial function in cancer progression and development. In this study, based on circRNA expression profile (GSE94591) analysis, a novel circRNA hsa_circ_0079480 was identified as being increased in AML. Herein, we identified that hsa_circ_0079480 was highly abundant and largely localized to the cytoplasm of circRNA molecules. Furthermore, hsa_circ_0079480 level was upregulated in BM samples obtained from AML patients. Remarkably, hsa_circ_0079480 inhibition was found to reduce advancement of AML by alleviating cell proliferation and stimulating cellular apoptosis.

An increasing number of studies demonstrated the interaction between circRNAs and miRNA, therefore regulating downstream target genes, which is a common method to regulate tumorigenesis [26, 27]. In this analysis, we predicted miRNA targets of hsa_circ_0079480 using Circular RNA Interactome and circBank tools. Among possible targets, we chose miR-654-3p for additional analysis due to their anti-tumor characteristics. For example, Yang et al. showed that miR-654-3p is reduced in liver cancer and hinders tumor cell growth and invasion *in vitro* [28]. Duan et al. demonstrated that lncRNA EMX2OS encouraged the progression of ovarian cancer cells by targeting the miR-654-3p/AKT3/PD-L1 pathway [29]. Jin et al. demonstrated that circRNA circHIPK3 is a prognostic biomarker in glioma development by modulating the miR-654/IGF2BP3 axis [30].

Herein, we also identified that miR-654-3p was decreased in AML, and low expression was linked to poor disease outcomes. Next, functional assays showed that miR-654-3p mimics suppressed growth and induced apoptosis of AML cells. Moreover, luciferase reporter and RIP assays further demonstrated that hsa_circ_0079480 could directly interact with miR-654-3p in AML. Thus, we demonstrated that hsa_circ_0079480 may have a role as a sponge of miR-654-3p in AML development.

HDGF, heparin-binding nuclear growth factor, was obtained from conditioned media of the hepatoma cell line Huh-7 [31]. Recently, some studies have demonstrated that HDGF serves an oncogenic function in many cancer types. For instance, Zhang et al. showed that high HDGF promoted bladder cancer cell development by stimulating the PI3K-AKT signaling axis [32]. Zheng et al. indicated that lncRNA AGAP2-AS1 promotes glioma cell progression by regulating the miR-15a/b-5p/HDGF/Wnt/β-catenin axis [33]. Though, the function of HDGF in AML remains unknown. In this analysis, we demonstrated that high HDGF expression in AML was related to poor OS in patients. Next, bioinformatics analysis suggested that HDGF serves as a possible target of miR-654-3p, and the binding between the two molecules was further confirmed by luciferase reporter assay. We also discovered that HDGF levels were inhibited after hsa_circ_0079480 silencing, and miR-654-3p inhibition rescued HDGF expression. Furthermore, functional rescue assays showed that the influence on cell viability and apoptosis stimulated by si-circ0079480 were hindered by miR-654-3p inhibition or HDGF overexpression. This information showed that hsa_circ_0079480 modulated AML cell development by regulating the miR-654-3p/HDGF pathway.

In summary, this analysis validated that hsa_circ_0079480 was highly expressed in AML, which promoted cell proliferation by upregulating HDGF expression via sponging miR-654-3p. Thus, the current findings provided an in-depth understanding of the basic research of circRNA intervention in AML in order to identify a new target for AML therapy.

## MATERIALS AND METHODS

### Patients’ tissues

Bone marrow (BM) samples from 12 AML patients were included in this analysis. Among the 12 samples, 7 were from patients with idiopathic thrombocytopenic purpura (ITP), which were regarded as negative controls (N). All tissues were acquired from The First People’s Hospital of Shangqiu. All participants in the present study provided informed consent, and the study was granted approval by ethics committee of the hospital.

### Cell culture and transfection

AML cell lines (MOLM-13 and AML-193) were bought from American Type Culture Collection (ATCC, Manassas, VA, USA) and grown using RPMI-1640 medium (Gibco, Waltham, MA, USA), which contains 10% fetal bovine serum (FBS; Gibco), in an incubator at 5% CO_2_ at 37° C.

siRNA that target hsa_circ_0079480 (si-circRNA), miR-654-3p mimics, miR-654-3p inhibitors, and negative controls [17] were acquired through GenePharma (Shanghai, China). The transfection was conducted through the use of Lipofectamine 2000 (Invitrogen, Carlsbad, CA, USA) as per established guidelines.

### RNA isolation and qRT-PCR

Total RNA was isolated from tissues and cell lines by utilizing TRIzol reagent (Invitrogen) as per manufacturer’s guidelines. Then, RNA was reverse-transcribed into cDNA through PrimeScript RT Reagent Kit (TaKaRa, China). qRT-PCR was conducted on an ABI 7500 instrument (ABI, Foster City, CA, USA) with SYBR Premix EX Taq Kit (TaKaRa). Relative gene expression was analyzed with 2^−ΔΔCt^ method. *GAPDH* and *U6* are internal controls. The primers included: hsa_circ_0079480-F, 5’-CAACCTCTGATTTTCAAGAAACC-3’ and hsa_circ_0079480-R, 5’-TTCACTTTTCAGCACTTCTTCAA-3’; miR-654-3p-F, 5’-CCGAGTATGTCTGCTGACCAT-3’ and miR-654-3p-R, 5’-CTCAACTGGTGTCGTGGA-3’.

### RNA fluorescence *in situ* hybridization (FISH)

The FITC-labeled hsa_circ_0079480 probes were designed and produced by Geneseed Biotechnology (Guangzhou, China). AML cells were grown on round coverslips, fixed, permeabilized in phosphate-buffered saline (PBS) with 0.5% Triton X-100, and dehydrated in ethanol. The FISH probes were diluted (1:50), denatured, balanced, and added to cells at 37° C overnight. After hybridization, the cells were labelled using DAPI-antifade for 10 min at room temperature. Finally, slides were sealed using rubber cement, placed in the dark for more than 20 min, and detected using a Leica microscope (Leica DM6000B, Switzerland).

### CCK-8 assay

Cell proliferation was evaluated utilizing Cell Counting Kit-8 (CCK-8) (Dojindo, Shanghai, China). Transfected cells/well (2 × 10^3^) were seeded in a 96-well plate. Post-incubation for 24, 48, 72, and 96 h, 10 μL CCK-8 medium was put into every well and placed for 4 h at 37° C. Absorbance was quantified at 450 nm by Absorbance Microplate Reader ELx808 (EnSight, USA).

### 5-Ethynyl-2’-deoxyuridine (EdU) assay

EdU assays were conducted utilizing a Cell-Light EdU DNA Cell Proliferation Kit (RiboBio, Guangzhou, China). Cells were then added into 96-well plates and treated with EdU for 2 h, followed by PBS washes and labelling with Apollo for 30 min. Subsequently, cells were fixed in 4% paraformaldehyde and labelled with DAPI (100 μL, 10 min). The images were taken using a fluorescence microscope (Olympus).

### Cell apoptosis assay

Cell apoptosis was measured utilizing the apoptosis detection kit (Sigma). 2 × 10^5^ AML cells per well were inoculated into 12-well plates. After 24 h treatment, cells were gathered and treated using Annexin V binding buffer, which was followed by labelling with Annexin V-FITC and 7AAD (Sigma). Subsequently, the percentage of apoptotic cells was assessed using flow cytometry (FACScan; BD Biosciences, Franklin Lake, NJ, USA) utilizing the CellQuest software (BD Biosciences).

### Dual-luciferase reporter gene assay

The wild-type (Wt) or mutant (Mut) vectors of hsa_circ_0079480 and HDGF were sub-cloned into pGL3 Luciferase Reporter Vectors (Promega). Next, plasmids were co-transfected using miR-654-3p mimics or negative controls (miR-NC). After 48 h, Dual-luciferase Reporter Assay System (Promega) helped evaluate luciferase reporter activities, as per established guidelines.

### RNA immunoprecipitation (RIP) assay

The relationship among hsa_circ_0079480 and miR-654-3p was assessed utilizing RIP assay through the EZ-Magna RIP kit (Millipore, Billerica, MA, USA), as per published instructions [18].

### Statistical analysis

Data was assessed by GraphPad Prism 6.0 and SPSS 21.0 software, and represented as mean ± SD of three individual experiments. Additionally, student’s t-test or one-way ANOVA was utilized to assess any variation among different groups. P-value < 0.05 represents significance.

## References

[1] Deschler B, Lübbert M. Acute myeloid leukemia: epidemiology and etiology. Cancer. 2006; 107:2099–107. 10.1002/cncr.22233 17019734

[2] Bray F, Ferlay J, Soerjomataram I, Siegel RL, Torre LA, Jemal A. Global cancer statistics 2018: GLOBOCAN estimates of incidence and mortality worldwide for 36 cancers in 185 countries. CA Cancer J Clin. 2018; 68:394–424. 10.3322/caac.21492 30207593

[3] Döhner H, Weisdorf DJ, Bloomfield CD. Acute myeloid leukemia. N Engl J Med. 2015; 373:1136–52. 10.1056/NEJMra1406184 26376137

[4] Appelbaum FR, Gundacker H, Head DR, Slovak ML, Willman CL, Godwin JE, Anderson JE, Petersdorf SH. Age and acute myeloid leukemia. Blood. 2006; 107:3481–85. 10.1182/blood-2005-09-3724 16455952 PMC1895766

[5] Jongen-Lavrencic M, Grob T, Hanekamp D, Kavelaars FG, Al Hinai A, Zeilemaker A, Erpelinck-Verschueren CA, Gradowska PL, Meijer R, Cloos J, Biemond BJ, Graux C, van Marwijk Kooy M, et al. Molecular minimal residual disease in acute myeloid leukemia. N Engl J Med. 2018; 378:1189–99. 10.1056/NEJMoa1716863 29601269

[6] Vo JN, Cieslik M, Zhang Y, Shukla S, Xiao L, Zhang Y, Wu YM, Dhanasekaran SM, Engelke CG, Cao X, Robinson DR, Nesvizhskii AI, Chinnaiyan AM. The landscape of circular RNA in cancer. Cell. 2019; 176:869–81.e13. 10.1016/j.cell.2018.12.021 30735636 PMC6601354

[7] Shang Q, Yang Z, Jia R, Ge S. The novel roles of circRNAs in human cancer. Mol Cancer. 2019; 18:6. 10.1186/s12943-018-0934-6 30626395 PMC6325800

[8] Meng S, Zhou H, Feng Z, Xu Z, Tang Y, Li P, Wu M. CircRNA: functions and properties of a novel potential biomarker for cancer. Mol Cancer. 2017; 16:94. 10.1186/s12943-017-0663-2 28535767 PMC5440908

[9] Zhou R, Wu Y, Wang W, Su W, Liu Y, Wang Y, Fan C, Li X, Li G, Li Y, Xiong W, Zeng Z. Circular RNAs (circRNAs) in cancer. Cancer Lett. 2018; 425:134–42. 10.1016/j.canlet.2018.03.035 29625140

[10] Yu T, Wang Y, Fan Y, Fang N, Wang T, Xu T, Shu Y. CircRNAs in cancer metabolism: a review. J Hematol Oncol. 2019; 12:90. 10.1186/s13045-019-0776-8 31484561 PMC6727394

[11] Wang J, Li H, Liang Z. circ-MYBL2 serves as a sponge for miR-361-3p promoting cervical cancer cells proliferation and invasion. Onco Targets Ther. 2019; 12:9957–64. 10.2147/OTT.S218976 31819492 PMC6877451

[12] Li X, Yang B, Ren H, Xiao T, Zhang L, Li L, Li M, Wang X, Zhou H, Zhang W. Hsa_circ_0002483 inhibited the progression and enhanced the taxol sensitivity of non-small cell lung cancer by targeting miR-182-5p. Cell Death Dis. 2019; 10:953. 10.1038/s41419-019-2180-2 31844042 PMC6915566

[13] Chen Q, Mang G, Wu J, Sun P, Li T, Zhang H, Wang N, Tong Z, Wang W, Zheng Y, Tian J, E M, Zhang M, Yu B. Circular RNA circSnx5 controls immunogenicity of dendritic cells through the miR-544/SOCS1 axis and PU.1 activity regulation. Mol Ther. 2020; 28:2503–18. 10.1016/j.ymthe.2020.07.001 32681834 PMC7646215

[14] Shang J, Chen WM, Liu S, Wang ZH, Wei TN, Chen ZZ, Wu WB. CircPAN3 contributes to drug resistance in acute myeloid leukemia through regulation of autophagy. Leuk Res. 2019; 85:106198. 10.1016/j.leukres.2019.106198 31401408

[15] Sun YM, Wang WT, Zeng ZC, Chen TQ, Han C, Pan Q, Huang W, Fang K, Sun LY, Zhou YF, Luo XQ, Luo C, Du X, Chen YQ. circMYBL2, a circRNA from MYBL2, regulates FLT3 translation by recruiting PTBP1 to promote FLT3-ITD AML progression. Blood. 2019; 134:1533–46. 10.1182/blood.2019000802 31387917 PMC6839953

[16] Shang J, Chen WM, Wang ZH, Wei TN, Chen ZZ, Wu WB. CircPAN3 mediates drug resistance in acute myeloid leukemia through the miR-153-5p/miR-183-5p-XIAP axis. Exp Hematol. 2019; 70:42–54.e3. 10.1016/j.exphem.2018.10.011 30395908

[17] Zhou X, Li J, Teng J, Liu Y, Zhang D, Liu L, Zhang W. Long noncoding RNA BSN-AS2 induced by E2F1 promotes spinal osteosarcoma progression by targeting miR-654-3p/SYTL2 axis. Cancer Cell Int. 2020; 20:133. 10.1186/s12935-020-01205-y 32351327 PMC7183609

[18] Wang J, Zhang JQ, Zhao XL, Lu JY, Weng ZM, Ding ZM, Yang FQ. Circular RNA DHX33 promotes Malignant behavior in ccRCC by targeting miR-489-3p/MEK1 axis. Aging (Albany NY). 2020; 12:14885–96. 10.18632/aging.103550 32717723 PMC7425503

[19] Dudekula DB, Panda AC, Grammatikakis I, De S, Abdelmohsen K, Gorospe M. CircInteractome: a web tool for exploring circular RNAs and their interacting proteins and microRNAs. RNA Biol. 2016; 13:34–42. 10.1080/15476286.2015.1128065 26669964 PMC4829301

[20] Liu M, Wang Q, Shen J, Yang BB, Ding X. Circbank: a comprehensive database for circRNA with standard nomenclature. RNA Biol. 2019; 16:899–905. 10.1080/15476286.2019.1600395 31023147 PMC6546381

[21] Huang HY, Lin YC, Li J, Huang KY, Shrestha S, Hong HC, Tang Y, Chen YG, Jin CN, Yu Y, Xu JT, Li YM, Cai XX, et al. miRTarBase 2020: updates to the experimentally validated microRNA-target interaction database. Nucleic Acids Res. 2020; 48:D148–54. 10.1093/nar/gkz896 31647101 PMC7145596

[22] Paraskevopoulou MD, Georgakilas G, Kostoulas N, Vlachos IS, Vergoulis T, Reczko M, Filippidis C, Dalamagas T, Hatzigeorgiou AG. DIANA-microT web server v5.0: service integration into miRNA functional analysis workflows. Nucleic Acids Res. 2013; 41:W169–73. 10.1093/nar/gkt393 23680784 PMC3692048

[23] Wong N, Wang X. miRDB: an online resource for microRNA target prediction and functional annotations. Nucleic Acids Res. 2015; 43:D146–52. 10.1093/nar/gku1104 25378301 PMC4383922

[24] Bao C, Wang J, Ma W, Wang X, Cheng Y. HDGF: a novel jack-of-all-trades in cancer. Future Oncol. 2014; 10:2675–85. 10.2217/fon.14.194 25236340

[25] Bai Y, Li X. Hsa_circ_0008285 facilitates the progression of cervical cancer by targeting miR-211-5p/SOX4 axis. Cancer Manag Res. 2020; 12:3927–36. 10.2147/CMAR.S244317 32547228 PMC7263883

[26] Liu R, Zhou M, Zhang P, Zhao Y, Zhang Y. Cell proliferation and invasion is promoted by circSERPINA3 in nasopharyngeal carcinoma by regulating miR-944/MDM2 axis. J Cancer. 2020; 11:3910–18. 10.7150/jca.42799 32328195 PMC7171482

[27] Chen G, Liu T, Yu B, Wang B, Peng Q. CircRNA-UBE2G1 regulates LPS-induced osteoarthritis through miR-373/HIF-1a axis. Cell Cycle. 2020; 19:1696–705. 10.1080/15384101.2020.1772545 32476580 PMC7469585

[28] Yang J, Zhang Z, Chen S, Dou W, Xie R, Gao J. miR-654-3p predicts the prognosis of hepatocellular carcinoma and inhibits the proliferation, migration, and invasion of cancer cells. Cancer Biomark. 2020; 28:73–79. 10.3233/CBM-191084 32176631 PMC12662334

[29] Duan M, Fang M, Wang C, Wang H, Li M. LncRNA EMX2OS induces proliferation, invasion and sphere formation of ovarian cancer cells via regulating the miR-654-3p/AKT3/PD-L1 axis. Cancer Manag Res. 2020; 12:2141–54. 10.2147/CMAR.S229013 32273754 PMC7102881

[30] Jin P, Huang Y, Zhu P, Zou Y, Shao T, Wang O. CircRNA circHIPK3 serves as a prognostic marker to promote glioma progression by regulating miR-654/IGF2BP3 signaling. Biochem Biophys Res Commun. 2018; 503:1570–74. 10.1016/j.bbrc.2018.07.081 30057315

[31] Kishima Y, Yoshida K, Enomoto H, Yamamoto M, Kuroda T, Okuda Y, Uyama H, Nakamura H. Antisense oligonucleotides of hepatoma-derived growth factor (HDGF) suppress the proliferation of hepatoma cells. Hepatogastroenterology. 2002; 49:1639–44. 12397753

[32] Zhang C, Chang X, Chen D, Yang F, Li Z, Li D, Yu N, Yan L, Liu H, Xu Z. Downregulation of HDGF inhibits the tumorigenesis of bladder cancer cells by inactivating the PI3K-AKT signaling pathway. Cancer Manag Res. 2019; 11:7909–23. 10.2147/CMAR.S215341 31692549 PMC6710542

[33] Zheng Y, Lu S, Xu Y, Zheng J. Long non-coding RNA AGAP2-AS1 promotes the proliferation of glioma cells by sponging miR-15a/b-5p to upregulate the expression of HDGF and activating Wnt/β-catenin signaling pathway. Int J Biol Macromol. 2019; 128:521–30. 10.1016/j.ijbiomac.2019.01.121 30684575

